# A comparison of weight gain between HIV exposed uninfected and HIV unexposed uninfected infants who received KMC at Chris Hani Baragwanath Academic Hospital

**DOI:** 10.3389/fped.2022.933968

**Published:** 2022-09-07

**Authors:** Leshata Abigail Mapatha, Firdose Lambey Nakwa, Mantoa Mokhachane

**Affiliations:** ^1^Department of Paediatrics and Child Health, Chris Hani Baragwanath Academic Hospital, School of Clinical Medicine, Faculty of Health Sciences, University of the Witwatersrand, Johannesburg, South Africa; ^2^Unit for Undergraduate Medical Education (UUME), School of Clinical Medicine, Faculty of Health Sciences, University of the Witwatersrand, Johannesburg, South Africa

**Keywords:** Kangaroo Mother Care (KMC), low birth weight (LBW), HIV, HIV exposed uninfected (HEU), HIV unexposed uninfected (HUU), weight gain (WG)

## Abstract

**Introduction:**

Kangaroo Mother Care (KMC) has been associated with improved growth in low birthweight infants and reduction in hypothermia, hypoglycaemia, apnoeas, sepsis, hospital stay, and mortality. The growth of HIV-infected children is poorer than those who are HIV-uninfected. There is paucity of data on weight gain in the HIV-exposed uninfected (HEU) infants compared to HIV-unexposed uninfected (HUU) infants receiving KMC.

**Aim:**

This study compared the weight gain of HEU and infants HUU from admission to the KMC ward until 12 months corrected age (CA) follow-up visit.

**Methods:**

Retrospective record review of the neonates admitted in KMC at Chris Hani Baragwanath Hospital over a 2-year period (2012–2013). The weight gain was assessed *via* weight velocity using the formula; weight/kg/day from admission to KMC to discharge, and g/ week at term, 3, 6 and 9- and 12-months (CA). The demographics were collected and analyzed using Statistica.

**Results:**

Seventy-seven (129/166) percent of the mothers were HIV negative. HIV negative mothers were younger (25.9 vs. 31.6 years; *p* = 0.000) and had fewer pregnancies (*p* = 0.02). There was no difference between the gestational age (30.3 ± 2.53 vs. 30.8 ± 2.88 weeks; *p* = 0.35) and birthweight (1,345 g ± 234 vs. 1,314 g ± 209; *p* = 0.47) between HEU and HUU. There were no differences in the weight gain (23.83 g ± 12.2 vs. 23.22 g ± 15.2; *p* = 0.83) in KMC. There was no differences in weight gain at the different follow-up time points between the two groups.

**Conclusion:**

Both HEU and HUU groups of infants showed reasonable weight gain despite maternal HIV status.

## Background

In 2015, 20.5 million low birth weight (LBW) babies were born worldwide, which was 14.6% of all births (1). An estimated 2.4 million newborn babies died in their 1st month of life in 2020. Nearly half (47%) of all under-five deaths in 2020 occurred during the neonatal period (1). Prematurity (60–80%) and asphyxia (23%) are regarded as the commonest causes of the early neonatal deaths, whilst infections are the commonest cause of later neonatal deaths (2, 3). The lack of sophisticated Neonatal Intensive Care Unit (NICU) facilities contributes to the poor survival rate of premature infants in developing countries (4). Strategies to improve neonatal survival including introduction of antenatal steroids, surfactant administration, newer modes of ventilation, strict aseptic measures and Kangaroo Mother Care (KMC) have significantly increased survival of LBW infants in high middle income countries (HMICs) such as South Africa (5).

Kangaroo Mother Care, also referred to as Skin-to-Skin Contact (SSC) has become an important intervention for caring for LBW infants, particularly in high middle income countries (HMIC) where there is a high mortality and morbidity rate (6). The introduction of KMC in HMIC reduces the incidence of hypothermia, hypoglycemia, apnea, sepsis and hospital stay and promotes overall growth of head circumference, length and weight in the premature infant which correlates with brain volume and better cognitive ability later in life (7).

The adoption of the 2013 WHO recommendation of lifelong antiretroviral therapy (ART) for all HIV positive pregnant women (known as PMTCT option B+) in January 2015 by the South African government has significantly reduced the number of HIV exposed and infected (HEI) infants, leading to an era of HIV exposed uninfected (HEU) infants (8).

Historically weight, height, and head circumference have generally been lower in HIV exposed children than their unexposed counterparts (9). Weight gain was found to be a good predictor of neurodevelopmental outcome especially in extremely low birth weight (ELBW) neonates (10). High demands of health care services to improve growth in LBW infants who are also HIV exposed in South Africa make KMC a cost-effective way to prevent neonatal mortality in the health facilities (11).

Growth faltering has been well-described in the HEU population from studies in Sub-Saharan Africa (12–15). Some studies reported poorer weight gain in females and breastfed infants (14). A few South African studies have found no differences in weight gain between the HEU group as compared to HUU infants (16, 17). Advanced HIV disease, being exposed to ART *in utero* and exposure to certain anti-retroviral (ARV) drugs adversely affects weight gain (13, 15, 16). HIV exposure and ARV drugs have been associated with impaired immunity and placental insufficiency *via* endothelial function impairment (13–15, 18, 19). HIV replicates in the placenta during pregnancy and may affect the T-cytokine profile in the placenta and thus restrict fetal growth and development which might contribute to LBW in HIV exposed infants (20). There have been varying reports on the timing and the effect of different ART regimens on growth (12, 14).

The aim of this study was to compare the weight gain in HEU and HUU infants from admission to the KMC ward until 12 months corrected age at follow-up visit.

## Methods

This study was conducted at Chris Hani Baragwanath Hospital, a tertiary hospital located in Soweto, South of Johannesburg, affiliated to the University of the Witwatersrand, South Africa. The unit had 175 neonatal beds and 25 KMC beds during the study period. The KMC ward is a facility for well-preterm born neonates who are waiting for weight gain and their mothers are encouraged to lodge in the ward to offer skin-to-skin care (SSC) to their babies throughout the day. During the study neonates weighing between 1,400 and 1,650 g and not requiring supplemental oxygen or intravenous fluids, were admitted to the KMC ward, provided their mothers were available to room-in with the baby. Mothers provided continuous SSC except when bathing or having meals, during which the babies were placed in the bassinets next to the mothers' bed. Length of stay was calculated from admission to KMC until discharge from KMC.

**Figure 1:**
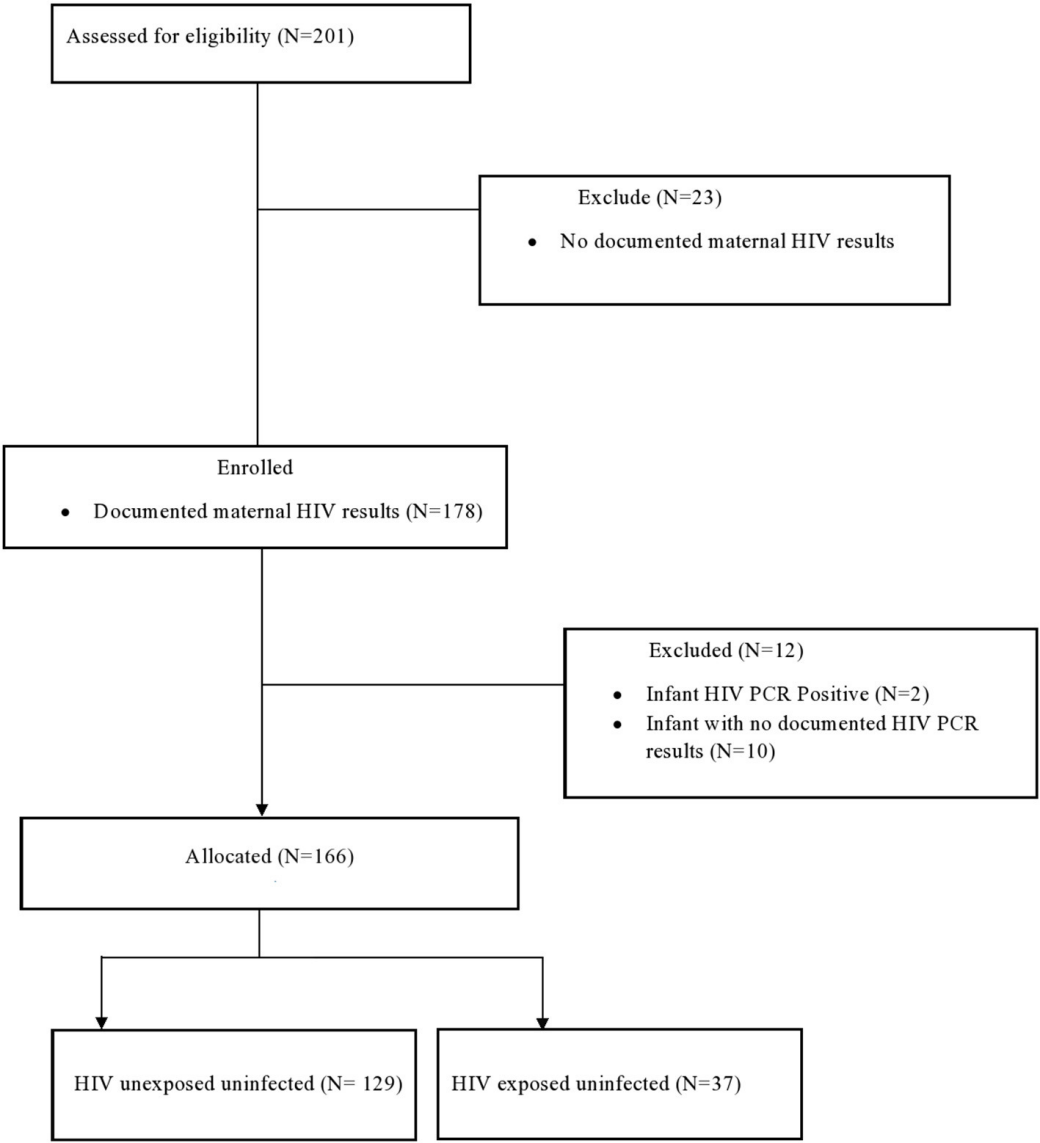
Flow diagram of neonates included in the study.

This is a retrospective study based on a review of medical records of LBW infants admitted in KMC over a 2-year period between 1st January 2012 and 31st December 2013. The study population included all HIV exposed and unexposed neonates weighing <1,650 g admitted to the KMC ward during the study period. All HIV positive neonates were excluded from the study (see Figure 1). Data was retrieved from medical records of the neonates that were admitted to the KMC and entered onto a datasheet. The data were entered onto an Excel Spreadsheet. Identifiers were kept separate from the neonatal and maternal demographics. A study number was assigned to the neonate, which was used to link the identifiers with the neonate's details. Neonatal records were divided into the HEU group and HUU group based on a documented maternal HIV status. All HIV exposed neonates (all neonates with positive maternal HIV test) had to have at least one documented polymerase chain reaction (PCR) result before being assigned to either the HEU or HUU group. At the time of the study, HIV in children was only tested at 6 weeks of age, 6 weeks post cessation of breast-feeding and 18 months of age (8).

Neonates were followed up at term [Corrected age (CA)–40 weeks], 3, 6, 9, and 12 months corrected age. To account for neonates not seen at the months mentioned above, a 1-month grace period on either side of the month was used for the infant's data to be included in the age group. For example, if the infant was seen at 4 months, the information was included in the 3-month age group. Information to be extracted from files included gestational age (GA), chronological age, corrected age (CA), birth weight, gender, HIV exposure, HIV status, mother's choice of feeds (breast fed or formula fed) supplements given (including FM85, a breastmilk fortifier), and the neonate's primary diagnosis (admitting diagnosis).

Each neonate's weekly weight was documented from date of admission into KMC ward until discharge. The discharge weight was 1,650 g at the time of the study in the KMC ward. Since participants were weighed daily during admission in KMC ward, only weights documented on Mondays and Thursdays were considered for the purpose of the study to avoid confusion. The median weight was calculated between the two weights. The patients were then weighed only on the day of follow up after discharge. To calculate the weight gained per week in g/week, the researcher used this formula: neonate's current weight in grams minus previous weight, divided by the number of weeks between the 2 weights. Adequate weight gain in KMC was set at 15 g/kg/day. For age 0–6 months this was 140–200 g/week and for those 6–12 months this was 85–140 g/week ^1^ (21).

### Data analysis

The data were analyzed utilizing Statistica (Statsoft USA, version 13). Categorical data were reported as numbers and percentages and continuous variables as means and standard deviations, if the data were normally distributed; if not normally distributed the data were represented as medians and interquartile ranges. To compare the HEU and the HUU groups, the Student's-*t* tests was used for continuous variables and the Chi-square test was used for categorical variables. A *p*-value < 0.05 was considered as significant.

### Ethical considerations

The study was approved by the Human Research Ethics Committee of the University of Witwatersrand (M180430). Permission to conduct the study was also obtained from the CHBAH hospital chief executive officer (CEO), the Pediatric and Neonatal Departmental Heads and National Department Health (NDoH).

## Results

A total of 201 neonates with accessible medical records were included in the study. Of the 201 mother infant pairs, 178 (89%) mothers had a documented HIV status. Of the 178 infants, two were PCR positive and 10 had no PCR results, therefore HIV PCR negative results were available on 166 infants. A total of 166 mother- infant pairs were included in the study (Figure 1).

The baseline maternal characteristics were similar between the two groups (Table 1). Of the 166 mothers who were involved in the study, 129 (78%) were HIV negative and 37 (22%) were HIV positive. Of the 37 HIV positive mothers, 31 (83.7%) received highly active anti-retroviral treatment (ART), and one did not receive ART and the Prevention of Mother to Child Transmission (PMTCT) records were unknown in 6 (16.2%). Thirty-two (84.2%) infants born to HIV positive mothers received Nevirapine (NVP), three (8.1%) received zidovudine (AZT), and 25 (67.6%) received oral trimethoprim-sulfamethoxazole (Bactrim). Eighty-two (49%) infants were born *via* Caesarian section and 55 (33%) born *via* normal vaginal delivery (NVD). The booking, employment and marital status of mothers did not differ by HIV-exposure group (Table 1).

**Table 1:** Maternal demographics of neonates admitted to the KMC ward.

**Characteristics**	**Total *N* (%)** ***N* = 166**	**HIV negative *N* (%)** ***N* = 129**	**HIV positive** ***N* (%) *N* = 37**	***p*-value**
Mean age (years)^*^	27.23 (±6.26)	25.9 (±2.5)	31.6 (±2.7)	0.00
Mean gravidity^*^	2.36 (±1.26)	2.17 (±1.17)	2.9 (±1.35)	0.02
Mean parity^*^	1.66 (±0.98)	2 (±1)	3 (±1)	0.00
**Mode of delivery**				
Cesarean section	82 (60)	65 (50)	17 (46)	0.83
NVD	55 (33)	40 (31)	15 (41)	
Missing	29 (18)	24 (19)	5 (13)	
**Booked**				
Yes	164 (99)	127 (98.5)	37 (100)	1.00
No	2 (1)	2 (1.5)	0	
**Employed**				
Yes	39 (24)	33 (26)	6 (16)	0.17
No	67 (40)	49 (38)	18 (49)	
Unknown	60 (36)	47 (36)	13 (35)	
**Marital status**				
Married	11 (7)	7 (5)	4 (11)	0.12
Single	114 (67)	89 (69)	25 (68)	0.28
Unknown	41 (26)	33 (26)	8 (21)	

* Mean (SD).

NVD, normal vaginal delivery; HIV, human immunodeficiency virus.

There were no significant differences between the gestational ages (30.3 ± 2.53 vs. 30.8 ± 2.88 weeks; *p* = 0.35) and birthweight (1,345.3 g ± 234 vs. 1 314.8 g ± 209.52; *p* = 0.47) in both groups. The majority of the infants involved in the study were female (*N* = 89, 54%). The incidence of RDS (*p* = 0.44) congenital pneumonia (*p* = 0.766), and necrotizing enterocolitis (*p* = 0.89) were not significant between the two groups. Eighty (63%) of the 129 HUU and 13 (35%) of the 37 HEU neonates had neonatal jaundice (NNJ) during their stay in the neonatal unit (*p* = 0.03). Length of stay in KMC was the same in both groups (13.2 ± 8.4 days in HUU vs. 14.0 ± 9.7 days in the HEU; *p* = 0.83; Table 2). There was no difference in mean weight gain in g/day in KMC and g/week at the different follow-up visits between the HUU and HEU (Table 3).

**Table 2:** Characteristics of neonates admitted to the KMC.

**Characteristics**	**Total**	**HUU** **mean (SD)**	**HEU** **mean (SD)**	***p*-value**
Birth weight in grams	1,338.49 ±228)	1,345.3 (±234)	1,314.8 (±209)	0.47
Gestational age in weeks	30.4 (±2.61)	30.3 (±2.53)	30.8 (±2.88)	0.35
**Apgar score**				
1 min	7 (±1.9)	7 (±2)	7 (±2)	0.44
5 min	9 (±1.13)	9 (±1)	9 (±1)	0.15
Female	89 (54)	70 (55)	19 (51)	0.625
RDS^*^	128 (78)	101 (79)	27 (73)	0.44
Congenital pneumonia^*^	17 (10)	14 (11)	3 (8)	0.76
NEC^*^	29 (17)	23 (18)	6 (16)	0.89
NNJ^*^	93 (56)	80 (63)	13 (35)	0.03
Weight at admission to KMC (grams)	1,408.9 (±1.46)	1,402.0 (±145.12)	1,434.8 (±151.11)	0.3
Weight at discharge from KMC (grams)	1,674.5 (±100.89)	1,673.6 (105.19)	1,677.6 (85.60)	0.83
Length of stay in KMC in days	13.3 (±8.7)	13.2 (±8.4)	14.0 (±9.7)	0.64

* N (%).

HUU, HIV Unexposed Uninfected; HEU, HIV Exposed Uninfected; RDS, respiratory distress syndrome; NEC, necrotizing enterocolitis; NNJ, neonatal jaundice; KMC, Kangaroo Mother Care.

**Table 3:** Growth velocity in KMC and at term, 3, 6, 9, and 12 months.

**Characteristic**	**HUU**	**HEU**	***P*-value**
	**Means (SD)**	**Means (SD)**	
Weight gain in KMC	*n* = 91	*n* = 24	0.83
g/day	23.83 (±12.2)	23.22 (±15.2)	
Term	*n* = 86	*n* = 24	0.44
g/week	205.4 (±74.7)	192.8 (±49.9)	
3 months	*n* = 71	*n* = 22	0.72
g/week	194.4 (±56.2)	189.6 (±49.7)	
6 months	*n* = 48	*n* = 14	0.91
g/week	173.5 (±46.6)	163.1 (±33.9)	
9 months	*n* = 26	*n* = 15	0.90
g/week	136.6 (±31.7)	135.5 (±21.3)	
12 months	*n* = 20	*n* = 7	0.81
g/week	117.9 (±22.6)	115.7 (±15.8)	

HUU, HIV Unexposed Uninfected; HEU, HIV Exposed Uninfected; KMC, Kangaroo Mother Care.

## Discussion

KMC has many benefits to the low-birth-weight infant including improvement in weight gain and haemodynamic stability. Infants in KMC feed more frequently and this further promotes overall maternal-infant bonding. In the growing group of HIV exposed infants, this form of intervention becomes even more necessary to implement as this group of infants is already vulnerable to infections and poor growth. In our study, both HUU and HEU had adequate weight gain from term to 12 months CA. In Kenya, growth decline was documented in an HEU group after 6 months (22). This finding is not consistent with most of the literature. In a cohort study of HEU children from the pre-ART era in Zimbabwe, it was shown that HEU children had a 23% higher chance of stunting than the HUU children at 12 months of age (23). This is similar to studies done in Uganda and Zambia which both showed lower weight- for- age and lower height -for- age in the HEU vs. HUU infants.

In a more recent study done in Zimbabwe, a larger number of HEU were born preterm and had low birth weight when compared to the HUU infants. In the same study, the mean weight- for- age and length- for- age were significantly lower in HEU compared to HUU infants from birth to 16 weeks (24). A study in Cape Town has reported on lower birth weights in HEU neonates (13). The duration of ART antenatally may be a factor as this may affect the placenta, despite no differences found dependent on the timing of exposure. More studies are to be conducted investigating the ART regimens and the timing of exposure (13). To the contrary, another South African study found no differences in birthweight in HEU compared to HUU neonates, and longitudinal growth was greater in formula fed infants (14). The lack of a significant difference in weight gain between the HEU and HUU was an important finding. Maternal drugs such as ART and HIV prophylactic drugs received by the HEU infants such as NVP, AZT and trimethoprim-sulfamethoxazole do not affect growth in the HEU group (25). However, some studies showed poor growth in the HEU infants due to *in utero* exposure to HIV and maternal ART (26).

In South Africa, severe advanced maternal disease had poorer growth patterns whilst exposure to ART was associated with better weight gain (14). Pre-pregnancy maternal ART was associated with stunting and poor weight gain in a Kenyan study (15). These differences in studies may be related to the different regimens that are available in the various countries. Ejigu et al. in an Ethiopian cohort reported slower growth in HEU neonates, with growth faltering related to earlier exposure to ART, maternal disease and the type of ART (12). Our study did not have information on the maternal CD4 count nor viral load or the duration of maternal ART. More prospective studies regarding the timing of ART, the combination of ART and maternal disease severity are warranted to document the effects on growth patterns in our setting.

The incidence of developing RDS, congenital pneumonia and NEC was similar in both groups. This is similar to a study done in British Columbia, Canada which showed similar incidences of infectious respiratory and non-respiratory illnesses in HUU and HEU neonates. In this particular study the HEU were prone to acquiring infections requiring NICU admission compared to their HUU counterparts (27). The results in our study only focused on co-morbidities at admission to the neonatal unit, prior to admission to the KMC ward and no differences were noted between the two groups. These prior comorbidities did not affect weight gain in the KMC ward as shown in Table 3.

A higher proportion of HUU neonates had neonatal jaundice. This finding is in keeping with a study done in a hospital in Malawi, which showed a higher incidence of jaundice in HIV unexposed neonates as compared to the HIV exposed neonates. One of the possible hypotheses for this finding as postulated by the authors is the use of drugs such as efavirenz, tenofovir, and lamivudine in pregnant and breast-feeding women as part of the prevention of mother-to-child transmission (PMTCT) programme in Malawi Efavirenz may act as a fetal liver enzyme inducer which helps in the conjugation of bilirubin thereby reducing the risk of jaundice in the HIV exposed infants (28).

South Africa is a poorly resourced HMIC country and thus most LBW infants do not have access to sophisticated neonatal care and equipment. This further highlights the important need for more KMC units in health care centers especially in the rural and semi-rural communities (7).

Maternal wellbeing is central to all aspects of infant's growth. Other factors which include nutritional, socioeconomic, health and environmental factors have an impact on childhood growth patterns. Although these factors were not assessed in this study, maternal height, body mass index (BMI) and education have been reported to contribute to growth faltering (15). An additional factor that may influence growth is family stress including other children left at home in poor socioeconomic circumstances and lack of support from hospital staff and family members (29). In Malawi maternal attitude to KMC was affected by mentorship, support, enthusiasm and training amongst health care workers and by family stress or support, especially by a lack of paternal support (30). These same factors also affected bonding between mother and infant. These factors are especially significant in mothers who are living with HIV due to the stigma associated with HIV (30).

The strength of our study is that it included a comparator group from the same community with similar socioeconomic status. Limitations of the study include that it was a single center study, and that we assessed growth using only weight gain at different ages and did not measure changes in length and head circumference. A further delineation of the maternal HIV status in terms of advanced disease, viral loads, CD 4 counts and ART regimens and when it was commenced were not investigated. The number of neonates and weights that were included at the different follow-up visits decreased over time.

## Conclusion

Patients in this study gained weight at a normal rate with no difference in weight gain between HEU and HUU infants. Interventions promoting KMC should be implemented in both HEU and HUU infants as KMC is simple, effective and can be implemented with minimal resources. Future studies should assess changes in length and head circumference as well as weight gain. The finding of an increased incidence of jaundice in HEU infants as compared to the HUU infants should be explored in future studies.

## Data availability statement

The original contributions presented in the study are included in the article/supplementary material, further inquiries can be directed to the corresponding author.

## Ethics statement

The studies involving human participants were reviewed and approved by Human Research Ethics Committee of the University of Witwatersrand. Written informed consent from the participants' legal guardian/next of kin was not required to participate in this study in accordance with the national legislation and the institutional requirements.

## Author contributions

All authors listed have made a substantial, direct, and intellectual contribution to the work and approved it for publication.

## Conflict of interest

The authors declare that the research was conducted in the absence of any commercial or financial relationships that could be construed as a potential conflict of interest.

## Publisher's note

All claims expressed in this article are solely those of the authors and do not necessarily represent those of their affiliated organizations, or those of the publisher, the editors and the reviewers. Any product that may be evaluated in this article, or claim that may be made by its manufacturer, is not guaranteed or endorsed by the publisher.

## Footnotes

^1^https://www.mayoclinic.org/healthy-lifestyle/infant-and-toddler-health/expert-answers/infant-growth/faq-20058037.
